# Nano-iron oxide accelerates growth, yield, and quality of *Glycine max* seed in water deficits

**DOI:** 10.3389/fpls.2022.992535

**Published:** 2022-09-09

**Authors:** Dipanjoli Baral Dola, Md. Abdul Mannan, Umakanta Sarker, Md. Abdullah Al Mamun, Tofazzal Islam, Sezai Ercisli, Muhammad Hamzah Saleem, Baber Ali, Oana Lelia Pop, Romina Alina Marc

**Affiliations:** ^1^Department of Agronomy, Faculty of Agriculture, Bangabandhu Sheikh Mujibur Rahman Agricultural University, Gazipur, Bangladesh; ^2^Department of Genetics and Plant Breeding, Faculty of Agriculture, Bangabandhu Sheikh Mujibur Rahman Agricultural University, Gazipur, Bangladesh; ^3^Institute of Biotechnology and Genetic Engineering, Bangabandhu Sheikh Mujibur Rahman Agricultural University, Gazipur, Bangladesh; ^4^Department of Horticulture, Faculty of Agriculture, Ataturk University, Erzurum, Turkey; ^5^College of Plant Science and Technology, Huazhong Agricultural University, Wuhan, China; ^6^Department of Plant Sciences, Quaid-i-Azam University, Islamabad, Pakistan; ^7^Department of Food Science, University of Agricultural Science and Veterinary Medicine, Cluj-Napoca, Romania; ^8^Food Engineering Department, Faculty of Food Science and Technology, University of Agricultural Science and Veterinary Medicine Cluj-Napoca, Cluj-Napoca, Romania

**Keywords:** foliar spray, seed yield, nano Fe_3_O_4_ particles, seed oil content, seed protein content, drought stress

## Abstract

Drought is one of the most destructive abiotic stresses that impact the growth, physiology, yield, and nutritional quality of seeds of crop plants. In modern agriculture, the use of nanoparticles can be beneficial due to their large surface area and higher potentiality to enter into the plant leaf during foliar application. This study aims to evaluate the effects of foliar spray containing varying doses (0, 100, and 200 ppm) of the nano-iron (Fe_3_O_4_) on the growth, physiology, yield, and seed nutritional quality of soybean under drought (40% of field capacity, FC) and well-watered (80% of FC) conditions. Leaf water status, chlorophyll content of leaves, the height of the plant, fresh leaf weight, fresh stem weight, fresh petiole weight, total dry weight, seed yield, and protein and oil content in soybean seeds were found to be suppressed by the applied drought stress. Under both drought (40% of FC) and controlled well-watered (80% of FC) conditions, the foliar application of nano-iron substantially improved the growth, physiology, yield, and quality of soybean seeds. The nanoparticles at 200 ppm increased soybean seed yield by 40.12 and 32.60% in drought and well-watered conditions, respectively, compared to the untreated plants. Furthermore, nano-iron increased the oil content of soybean seeds by 10.14 and 7.87% under drought and well-watered conditions, respectively, compared to the untreated control. Our results indicate that the application of nano-iron improved drought tolerance, yield, and seed quality of soybean, so exogenous foliar sprays of 200 ppm Fe_3_O_4_ were more effective than the other treatments in enhancing drought tolerance and can be utilized to reduce losses caused by drought stress in soybean-growing areas.

## Introduction

Soybean (*Glycine max* L.) is an annual self-pollinated diploid legume crop plant belonging to the family Fabaceae. It is one of the most prevalently grown and used oilseed crops and a rich source of plant proteins. In Bangladesh, both annual soybean production and imports are increasing gradually to meet the feed requirements for the livestock, poultry, and fisheries sectors, and soybean oil for human consumption (Islam et al., 2022). However, soybean is one of the most competitive crops for the farmers of Bangladesh, as it is mainly cultivated in marginal lands, especially in the drought-prone *char* areas and coastal land areas (Khan, 2013).

Abiotic stress factors, such as drought and salinity, impair the productivity of crops (Sarker and Oba, 2019b; Hussain et al., in press) through the generation of reactive oxygen species (ROS), which induce oxidative damage and osmotic stress (Sarker and Oba, 2018a, 2020b), and cause many physiological and biochemical changes in the plants, including membrane, DNA, and protein damage, nutrient imbalance (Sarker and Oba, 2018b,c; Ma et al., 2022), and decrease in photosynthetic activities and changes in color pigments (Sarker and Oba, 2018d,e; Sarker et al., 2018). Drought is one of the most detrimental abiotic stresses faced by crop plants, which is responsible for reducing seed growth, yield, and nutritional quality (Naumann et al., 2018). Climate change has become one of the most serious issues globally and is responsible for the frequent and intense drought conditions in recent years (Ali et al., 2022; Hossain et al., 2022). Due to this abiotic stress factor, about 25% of agricultural production is getting affected (Fathi and Tari, 2016; Ahmad et al., 2022). Among all the abiotic factors, drought is considered the most devastating, affecting different molecular, biochemical, physiological, morphological, and ecological traits, and processes involved in all growth and developmental stages. Due to this water-limited condition, plant yield and quality are deteriorating exponentially, causing huge losses in soybean yield (Basal and Szabó, 2020).

Nanoparticles can be used efficiently as a fertilizer to enhance the tolerance over abiotic stress, such as drought (Kashyap et al., 2017). The mixture of iron nanoparticles (Fe NPs) and salicylic acid has resulted in drought conditions in strawberry (*Fragaria* × *ananassa*) plants (Kashyap et al., 2016). Zainab et al. (2021) reported that drought stress decreased oil (10.30%) and grain protein yield (24.90%) of sesame. After using iron nano-fertilizer, soybean yield was found to be increased (Sheykhbaglou et al., 2010). Jaberzadeh et al. (2013) observed that about 23.3% of grain yield was increased by the foliar application of 2% nano-iron.

The most significant characteristics of nanoparticles (NPs) are their larger surface area, particle shape, size, tunable pore size, and reactive potentiality (Khan et al., 2019). In plants, NPs target the cellular organelles (Zeljka et al., 2014) and play a role in increasing the bioavailability of plant nutrients (Meena and Aravinda, 2017) and improving the product quality (Vuong, 2019). Having a particle size of only <100 nm along with a higher surface area, nano-fertilizers can easily penetrate the plant leaves, which ensures maximum reactivity and results in the higher availability of nutrient content and nutrient use efficiency of the plant (Mahanta et al., 2019). Considering the unique features and versatile functions of plants, nanoparticles are considered promising tools for promoting sustainable agriculture in changing climate conditions (Mittal et al., 2020). This study aimed to evaluate the effects of foliar application of Fe_3_O_4_ nanoparticles on the growth, yield, and seed quality of soybean under drought-stressed conditions.

## Materials and methods

### Experimental site

The pot experiment was carried out under a semi-controlled environment in poly house conditions in the Department of Agronomy, Bangabandhu Sheikh Mujibur Rahman Agricultural University (BSMRAU), Bangladesh, from 2019 to 2020. The experimental site was situated in a subtropical climatic zone. The experimental site is the center of the Madhupur tract (AEZ 28) (24° 5′ 23″ N and 90° 15′ 36″ E) which is located 8.4 m above the mean sea level. The day and night temperatures were 28.5 ± 1.6 and 13.6 ± 1.3°C, respectively, in the poly house.

The sandy loam soil was used throughout the experiments (53.12% sand, 33.12% silt, and 13.76% clay) with a pH of 6.71. The soil organic carbon, available P, total N, exchangeable K, CEC, and EC were 0.59%, 0.07 mg 100 g^−1^, 0.06%, 0.76 cmol kg^−1^ dry soil, 12.85 cmol kg^−1^ dry soil, and 0.03 dS m^−1^, respectively. At field capacity (FC), the soil holds about 28% moisture. The mixture of soil and cow dung at a ratio of 4:1 was used in every plastic pot. The length of those pots was 30 cm, and the diameter was 24 cm, which was filled up with 11 kg of air-dried soil.

### Plant materials and nanoparticle solution

The soybean variety known as BU soybean-1 was used in this experiment. It is a high-yielding variety developed and released by Bangabandhu Sheikh Mujibur Rahman Agricultural University. For the preparation of the nano-iron solution, nanopowder of Fe_3_O_4_ (II, III) oxide with 97% trace metals was used. The particle size of the powder was 50–100 nm [scanning electron microscopy (SEM)]. The powder was purchased from Sigma- Aldrich. In 25 g of the product, only 1,458.8 ppm was constituted by trace metals. The chemical composition of the iron nanoparticle is presented in Table 1 (Sigma–Aldrich, 2016).

**Table 1:** Elements of trace metal of iron nano-Fe_3_O_4_ (Sigma–Aldrich, 2016).

**Elements**	**Concentration (ppm)**	**Percent**
Aluminum (Al)	227.2	0.02272%
Boron (B)	0.8	0.00008%
Barium (Ba)	1.6	0.00016%
Calcium (Ca)	241.4	0.02414%
Cadmium (Cd)	10.3	0.00103%
Chromium (Cr)	43.5	0.00435%
Copper (Cu)	18.3	0.00183%
Potassium (K)	11.7	0.00117%
Magnesium (Mg)	76.1	0.00761%
Manganese (Mn)	732.3	0.07323%
Molybdenum (Mo)	1.8	0.00018%
Sodium (Na)	58.9	0.00589%
Nickel (Ni)	34.9	0.00349%

To prepare 100 ppm nano-Fe_3_O_4_ solution, 100 mg of this powder was added to 1 L of distilled water. Similarly, 200 ppm of the nano-Fe_3_O_4_ solution was prepared by adding 200 mg of the Fe_3_O_4_ powder to 1 L of deionized water. Both solutions were heated at a temperature of 60°C for 16 h on a magnetic stirrer with a hot plate. After that, a sonication bath was given to both the solutions with continuous vibration to mix all the particles into the water homogenously, so that the solution could penetrate through the plant leaves effortlessly during the application (Sandhya et al., 2021). Then, these solutions were stored in a plastic bottle at room temperature. The required amount of solution was taken in a hand sprayer during the application of the solution to the plant.

### Treatments and cultural practices

Ten healthy seeds were sown maintaining uniform spacing in each pot. After sowing, minimal irrigation was applied using a beaker to maintain uniform germination. Thinning was done during the appearance of the first two-leaf stage, and six uniform and healthy plants were placed in each pot. Uniform application of 0.32 g of urea, 0.933 g of triple superphosphate (TSP), and 0.64 g of muriate of potash (MOP) was carried out in each pot, which corresponds to 80, 205, and 128 kg of urea, TSP, and MOP per hectare, respectively (Fertilizer Recommendation Guide, 2018). The pots were investigated regularly to identify the moisture level of the soil by using a portable digital moisture meter (POGO Soil Sensor II, Stevens, USA). At the trifoliate stage (14 days after sowing), the required amount of water was added regularly to maintain the field capacity to 80% in nine pots which are derived from well-watered (Control) plants, and the other nine pots were kept in water-stressed condition by having 40% field capacity (Drought) throughout the growing season. The experiment consisted of two factors: Factor A [well water (Control) and Drought] and Factor B (three doses of nano-Fe_3_O_4_). At the V2 stage (7 days after drought imposition), the control and drought-treated plant leaves were sprayed with water (0 ppm nano-Fe_3_O_4_), 100 ppm nano-Fe_3_O_4_ solution, and 200 ppm nano-Fe_3_O_4_ solutions through a hand sprayer. The whole plant was fully sprayed once in 2-week intervals, and it was performed four times in the whole growing season. The experiment was laid out in a factorial randomized complete block design (RCBD) with three replications.

### Growth and agronomic trait measurement

At the flowering stage (15 days after first spraying), three plants were harvested from each pot. Morphological growth-related parameters, such as the height of plants, leaf, stem, total fresh weight, and total dry weight, were measured. At the physiological maturity stage, the remaining three plants from each pot were harvested, and seed yield/plant and data of other yield contributing characters, that is, number of pods/plant, number of seeds/pod, and 100-seed weight, were also recorded. The harvested seeds of each treatment were used for measuring protein and oil content.

### Estimation of chlorophyll

Fully developed leaves located at the top of the plant were sampled after 15 days of first spraying (flowering stage), and the chlorophyll content was estimated according to Sarker and Oba (2019a, 2020a); Sarker et al. (2020). 20 mg of fresh leaf samples were collected in vials containing 20 ml of acetone (80%) and covered with aluminum foil and then stored in the dark for 72 h. The reading was taken at 663 nm and 645 nm using a double beam spectrometer (model 200-20), and chlorophyll *a* and *b*, and total chlorophyll were calculated using the following formula:

Chlorophyll *a* (mg/g fresh weight) = [12.7 (D663) – 2.69 (D645)] × [V/100 × W]

Chlorophyll *b* (mg/g fresh weight) = [22.9 (D645) – 4.68 (D663)] × [V/100 × W]

Total chlorophyll (mg/g fresh weight) = [20.2 (D645) – 8.02 (D663)] × [V/100 × W]

where D (663, 645) = Optical density of the chlorophyll extract at a wavelength of 663 and 645 nm, respectively.

V = Final volume (ml) of the 80% acetone with chlorophyll extract.

W = Weight of fresh leaf sample in g.

### Measurement of water status

After 15 days of first spraying, samples of fully developed green upper leaves were taken, and their weights were immediately recorded as fresh weight (FW). The leaves were then soaked in distilled water in a dark room for 24 h at room temperature. The next day, the turgid weight (TW) of these leaves was measured after gently wiping with a paper towel to remove excess water from the surface of the leaves. Finally, the leaves were dried for 48 h at 72°C, and the dry weight (DW) was recorded. Relative water content (RWC) and water saturation deficit (WSD) were measured according to the formula of Manette et al. (1988).


$$\text{RWC }(\text{\%})\;=\frac{\text{FW }-\text{ DW}}{\text{TW }-\text{ DW}}\times 100,\text{ WSD }=\frac{\text{TW }-\text{ FW}}{\text{TW }-\text{ DW}}\times 100$$

### Protein content

The protein content of the soybean seed was estimated by the Kjeldahl method according to the description given in VELP Scientifica (2015). About 0.5 g of seed powder sample was placed into a 250-ml test tube. Each sample was treated with copper sulfate (CuSO_4_) and potassium sulfate (K_2_SO_4_) in a 1:9 ratio for digestion. Then, 10 ml of 96–98% concentrated sulfuric acid (H_2_SO_4_) was added. The samples were digested chronologically for 10 min at 150°C, 10 min at 250°C, 15 min at 350°C, and 60 min at 420°C. Then, the test tubes were allowed to cool down at 50–60°C. The distillation and titration were done for 4 min using 0.2 N hydrochloric acid, 35% sodium hydroxide, 10 ml of bromocresol green solution, 7 ml of methyl red, and 4% boric acid (40 g boric acid in 960 ml of distilled water). Finally, after each titration cycle, the percentage of nitrogen and protein was shown automatically on the screen of the VELP SCIENTIFICA UDK 169 automatic distillation and titration system.

### Oil content

The oil content of soybean seeds was measured with the Soxhlet apparatus using the method described by Edward (1971). The powdered seed samples were dried and weighed in a thimble plugged with fat-free cotton. Then, the sample was dropped into the fat extraction tube of the Soxhlet apparatus. After that, an additional 75 mL of anhydrous ether was poured into the sample, and the sample was extracted for 16 h in a water bath. At the end of the extraction process, the thimble was removed, and ether was distilled off from the solution. The thimble-containing sample was saved for the estimation of crude fiber content. Then, the sample was poured into a weighed beaker, and the flask was rinsed thoroughly with ether. At 100°C, the sample was dried for 1 h, cooled, and weighed. The difference in the weights corresponded to the ether-soluble material present in the sample.


$$\%\;Crude\;fat\;(Oil)=\frac{Weight\;of\;ether\;soluble\;material}{Weight\;of\;sample}\times 100$$

### Statistical analysis

The CropStat statistical software version 7.2 was used to analyze the recorded data. Mean values of all treatments were compared using the least significant difference (LSD) test at a 5% significance level.

## Results

### Agronomic traits

The morphological and growth parameters of all soybean samples increased exponentially with the foliar application of 100 and 200 ppm Fe_3_O_4_ nanoparticles in both well-watered and drought stress conditions. Among both treatments, foliar application of 200 ppm nano-Fe_3_O_4_ fertilizer provides maximum growth in both conditions. After the observation of both drought and control (well-watered) conditions, a remarkable decrease in plant height, fresh leaf weight, fresh stem weight, total fresh weight, and total dry weight by 15.77, 44.57, 57.57, 48.3, and 26.34%, respectively, was witnessed as the result of drought conditions (Tables 2, 3). Specifically, plant height had been increased by 21.24 and 20.39% after the application of 200 ppm nano-Fe_3_O_4_ in drought and well-watered conditions, respectively, when compared to the plant samples not treated with a nano-fertilizer. However, the fresh leaf weight, fresh stem weight, total fresh weight, and total dry weight were enhanced significantly by 31.18, 45.64, 32.57, and 48.48%, respectively, under drought stress when 200 ppm nano-Fe_3_O_4_ fertilizer was used to treat the plants. In addition, these similar parameters exhibited an increase of 8.75, 26.06, 13.82, and 20.09%, respectively, after treating the plants with 200 ppm nano-Fe_3_O_4_ fertilizer under well-watered conditions. According to the observation, the effect of nano-fertilizer was always positive in both (drought and well-watered) conditions. More concisely, in drought conditions, the morphological growth of the nano-fertilizer-treated soybean plants was noticeably higher relative to that observed in well-watered conditions.

**Table 2:** Effect of nano-Fe_3_O_4_ fertilizers on plant height and fresh leaf weight of soybean after 15 days of first spraying (flowering stage) under well-watered and drought conditions.

**Nano iron (ppm)**	**Plant height (cm)**		**Leaf fresh weight/plant (g)**	
	**Control**	**Drought**	**Control**	**Drought**
0	26.83 b	22.60 c	13.71 a	7.60 c
100	31.37 a	25.90 b	14.39 a	9.12 bc
200	32.30 a	27.40 b	14.91 a	9.97 b
CV (%)	4.0		8.5	

Mean values followed by diverse letters differ significantly by LSD at p < 0.05.

**Table 3:** Effect of nano-Fe_3_O_4_ fertilizers on fresh stem weight, total fresh weight, and dry weight of soybean after 15 days of first spraying (flowering stage) under well-watered and drought conditions.

**Nano-iron (ppm)**	**Stem fresh weight/plant (g)**		**Total fresh weight/plant (g)**		**Total dry weight/plant (g)**	
	**Control**	**Drought**	**Control**	**Drought**	**Control**	**Drought**
0	5.68 b	2.41 c	19.39 b	10.01 d	2.24 a	1.65 b
100	6.77 ab	3.34 c	21.16 ab	13.06 c	2.53 a	2.12 ab
200	7.16 a	3.51 c	22.07 a	13.27 c	2.69 a	2.45 a
CV (%)	12.2		7.3		14.4	

Mean values followed by diverse letters differ significantly by LSD at p < 0.05.

### Physiological traits

#### Chlorophyll content

The water stress condition in soybean plants was also responsible for the declining trend of chlorophyll content in the plant leaves. Due to this stressed condition, a decrease of 22.88% chlorophyll *a*, 14.58% chlorophyll *b*, and 19.16% chlorophyll content in soybean leaves was noticed (Table 4) when compared to the control plants. The chlorophyll content increased significantly after the foliar application of nano-Fe_3_O_4_ fertilizers, and hence is an appropriate strategy to overcome the harmful effects of water deficiency. Between both treatments, plants treated with 200 ppm Fe_3_O_4_ nano-fertilizer show the maximum increased value of chlorophyll content. As a result of the foliar application of nano-fertilizer in drought conditions, the levels of chlorophyll *a*, chlorophyll *b*, and total chlorophyll were increased to 24.58, 36.46, and 29.91%, respectively, compared to the plants that were not treated. On the other hand, after the exogenous foliar application of Fe_3_O_4_ nano-fertilizer in controlled conditions, chlorophyll *a*, chlorophyll *b*, and total chlorophyll content showed an increment of 12.41, 20, and 15.69%, respectively, relative to the untreated plants. However, the percentage of the increasing trend in the well-watered condition was not as high as that observed under the drought condition.

**Table 4:** Effect of nano-Fe_3_O_4_ fertilizers on chlorophyll *a*, chlorophyll *b*, and total chlorophyll content of soybean leaf after 15 days of first spraying (flowering stage) under well-watered and drought conditions.

**Nano iron (ppm)**	**Chlorophyll** ***a*** **(mg/g FW)**		**Chlorophyll** ***b*** **(mg/g FW)**		**Total chlorophyll (mg/g FW)**	
	**Control**	**Drought**	**Control**	**Drought**	**Control**	**Drought**
0	1.45 ab	1.18 b	1.10 b	0.96 b	2.55 b	2.14 c
100	1.54 ab	1.36 b	1.23 ab	1.18 ab	2.77 ab	2.54 bc
200	1.63 a	1.47 ab	1.32 a	1.31 a	2.95 a	2.78 ab
CV (%)	7.0		6.5		5.3	

Mean values followed by diverse letters differ significantly by LSD at p < 0.05. FW = Fresh weight.

#### Leaf water status

**Figure 1:**
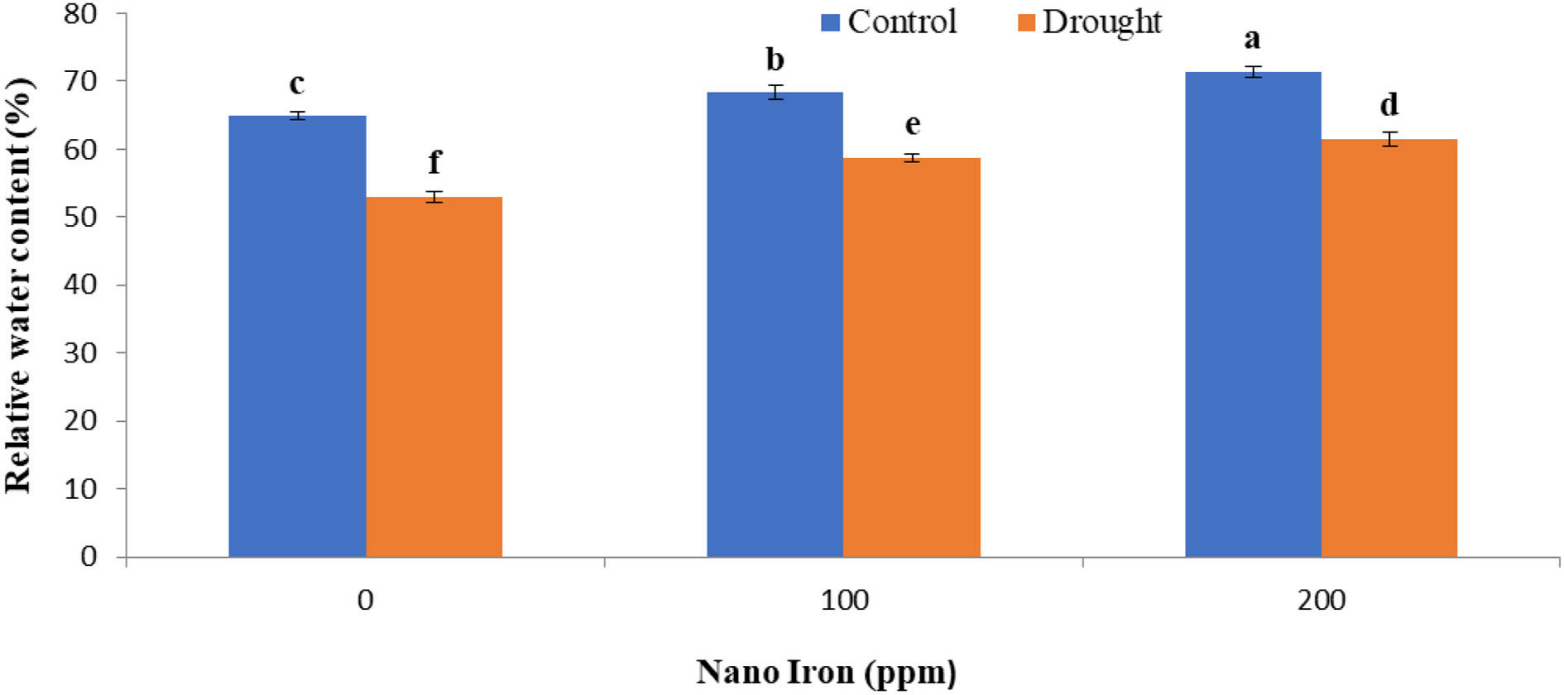
Effect of nano-Fe_3_O_4_ fertilizers on relative water content (RWC) of soybean after 15 days of first spraying (flowering stage) under well-watered and drought conditions. Bars indicate (±SE). Different letters in the bars indicate significantly differ at *p* < 0.05 by LSD.

**Figure 2:**
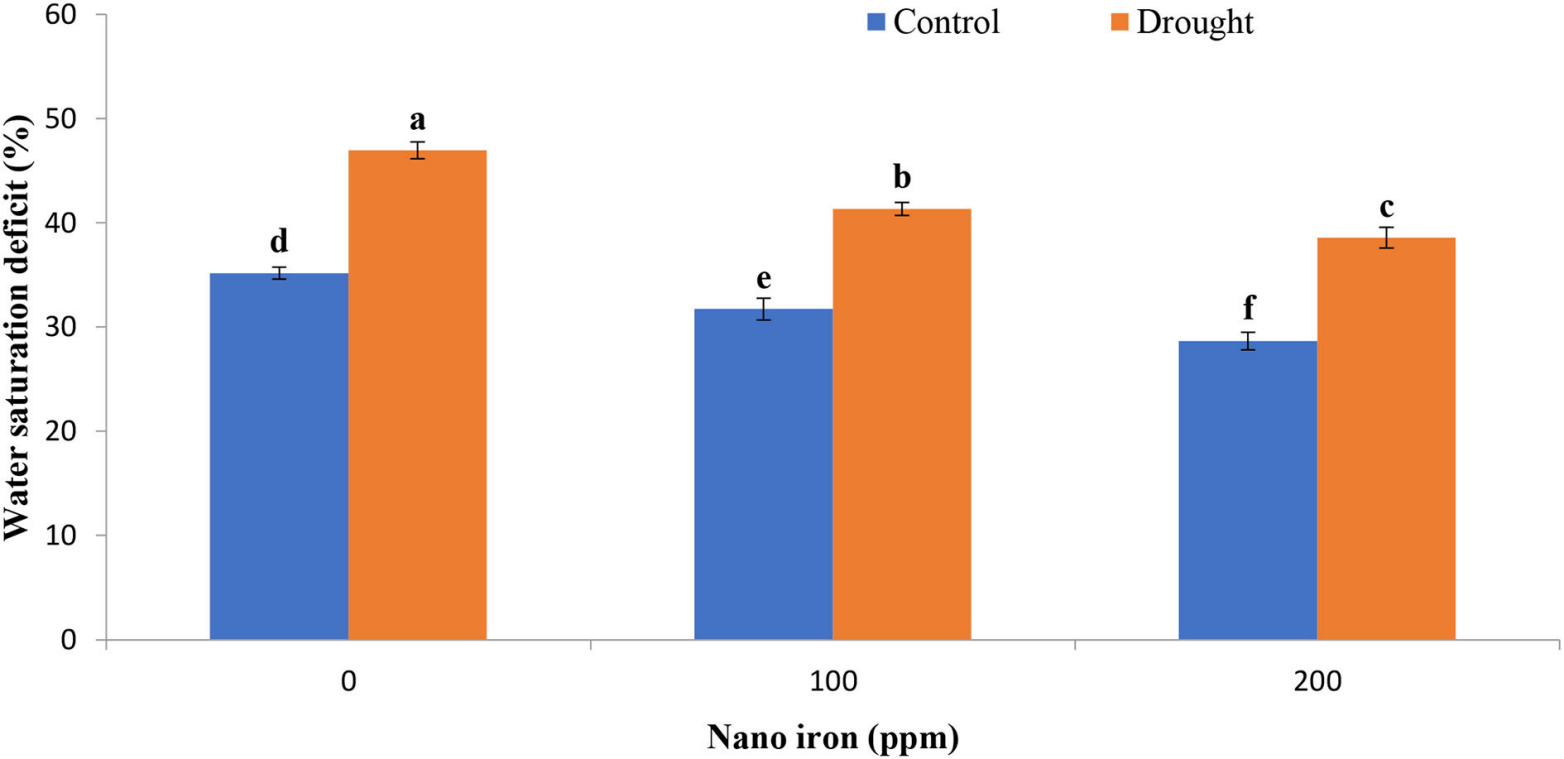
Effect of nano-Fe_3_O_4_ fertilizers on water saturation deficit (WSD) of soybean after 15 days of first spraying (flowering stage) under well-watered and drought conditions. Bars indicate (±SE). Different letters in the bars indicate significantly differ at *p* < 0.05 by LSD.

The relative water content (RWC) of soybean plants was severely diminished under the drought stress by 22.21% relative to the plants grown in well-watered conditions (Figure 1). The treatment of plants with different concentrations of nano-Fe_3_O_4_ fertilizer significantly suppresses the devastating effect of drought stress. As a result of the exogenous spraying of 200 ppm nano-Fe_3_O_4_ fertilizer on the leaf surface of drought-affected soybean plants, relative water content had been alleviated by 15.8 and 10.04% in well-watered plants when compared to the plants that were not treated. Conversely, the lack of adequate water content increased the saturation deficit (WSD) of plants by 33.49% when compared to those grown in the control conditions (Figure 2).

### Yield and yield contributing characters

The detrimental effect of drought has also been observed in soybean yield and yield contributing characteristics. Our study observed that the number of pods per plant, number of seeds per pod, and 100-seed weight had descended by 37.22, 34.19, and 55.34%, respectively, under drought stress when compared to the well-watered condition (Table 5). Similar to the growth parameters, the application of 200 ppm nano-Fe_3_O_4_ solution also increased the number of pods per plant by 11.74%, the number of seeds per pod by 43.27%, and 100-seed weight by 14.80% compared to the untreated plants in controlled condition. The response of most of the yield parameters of soybean plants due to the foliar spray of the intended treatment was extensively higher under drought stress than in the controlled condition. In the drought conditions, the number of pods per plant, number of seeds per pod, and 100-seed weight were alleviated by 28.71, 20.00, and 29.14%, respectively, in the untreated plants. Considering the seed yield per plant, drought stress caused a drastic reduction in the yield by 141% compared to the well-watered condition. Exogenous foliar spraying of 200 ppm Fe_3_O_4_ nano-fertilizer escalated the yield by 32.60% in well-watered conditions and about 40.12% in drought conditions compared to the other untreated plants (Table 5).

**Table 5:** Effect of nano-iron fertilizer on number of pods, number of seeds, 100-seed weight, and yield of soybean under well-watered and drought conditions.

**Nano iron (ppm)**	**Number of pods per plant**		**Number of seeds per pod**		**100-seed weight (g)**		**Yield/plant (g)**	
	**Control**	**Drought**	**Control**	**Drought**	**Control**	**Drought**	**Control**	**Drought**
0	55.44 b	40.40 d	2.08 bc	1.55 c	10.61 b	6.83 d	12.30 c	5.11 e
100	59.34 a	47.85 c	2.42 b	1.77 c	11.85 a	7.54 d	14.72 b	6.03 de
200	61.95 a	52.00 b	2.98 a	1.86 c	12.18 a	8.82 c	16.31 a	7.16 d
CV (%)	4.1		10.3		6.8		8.2	

Mean values followed by diverse letters differ significantly by LSD at p < 0.05.

### Seed protein and oil content

The fundamental nutritional value of soybean can be attributed to its protein and oil content. In the case of protein content, drought stress does not hamper this nutrient value. An increasing trend was observed in the protein content of soybean seed when nano-iron was applied in different concentrations under well-watered and drought conditions (Table 6). The oil content of soybean seeds considerably confronts the destructive effect of drought stress. Due to drought stress, oil content had decreased by 38% relative to the controlled condition. In drought conditions, nano-fertilizer improved the oil content of soybean by 10.14% in comparison to the plants which were not treated. The oil content of soybean seeds was also increased by 7.87% in well-watered conditions due to the foliar application of nano-Fe_3_O_4_ fertilizer when compared to the untreated condition (Table 6).

**Table 6:** Effect of nano-Fe_3_O_4_ fertilizers on protein content and oil content of soybean under well-watered and drought conditions.

**Nano iron (ppm)**	**Protein content (%)**		**Oil content (%)**	
	**Control**	**Drought**	**Control**	**Drought**
0	30.39 c	35.96 a	15.51 b	11.24 d
100	31.96 bc	36.73 a	16.06 ab	11.96 cd
200	32.62 c	36.92 a	16.73 a	12.38 c
CV (%)	2.9		3.6	

Mean values followed by diverse letters differ significantly by LSD at p < 0.05.

### Interrelationship between studied treatments and evaluated traits

**Figure 3:**
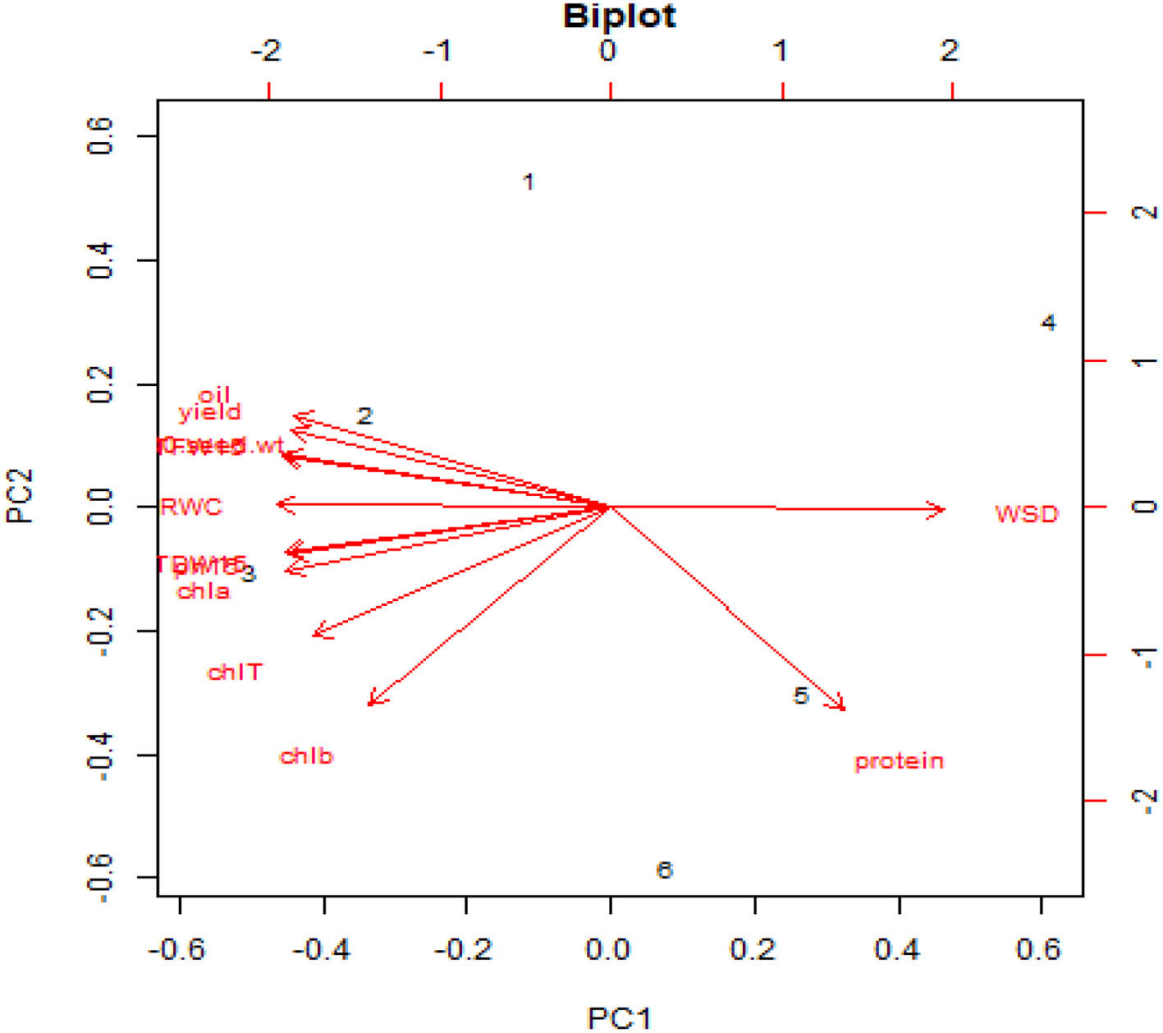
Biplot of PC1 vs. PC2.

The interrelationship between the evaluated treatments and traits was determined by performing principal components (PC) analysis (Figure 3). Most variations had been found in the first two PCs, and the approximate cumulative value was 99% (86.4% by PC_1_ and 12.6% by PC_2_). They were implicated in building the PC biplot. It is composed of six treatments: untreated well-watered (W + F0) and drought-stressed (D + F0) treatments, foliar application of nano-fertilizer 100 and 200 ppm dose of iron nano-fertilizer in well-watered conditions (W + F1 and W + F2), and foliar application of nano-fertilizer 100 and 200 ppm dose of iron nano-fertilizer in drought-stressed conditions (D + F1 and D + F2). The well-watered condition was located on the extremely negative side of the PC_1_, whereas the drought-stressed condition was located on the positive side.

Moreover, the foliar application of 100 and 200 ppm nano-iron solution in well-watered conditions was situated on the negative side of PC_1_, while the foliar application of 100 and 200 ppm nano-iron solution in drought-stressed conditions was found on the positive side. Additionally, a strong positive relationship can be identified for parallel vector traits or the traits that remained close to each other, and the traits that situated approximately opposite to each other indicated a negative correlation. A strong positive relationship was noticed between plant height (phT), total fresh weight (TFW), total dry weight (TDW), chlorophyll *a* (Chla), chlorophyll *b* (Chlb), total chlorophyll (ChlT), 100-seed weight, yield, oil content, and relative water content (RWC). On the other hand, the yield and contributing traits showed a negative interaction with water saturation deficit (WSD) and protein content.

## Discussion

As a result of the application of nano-fertilizer, the vegetative growth of soybean plants was observed to alleviate considerably compared to the plants grown in the well-watered condition. Other researchers have also shown that the growth rates and germination percentages of various crops can be improved by the application of Fe NFs relative to the control and/or synthetic Fe sources. Srivastava et al. (2014) found that the growth of spinach (*Spinacia oleracea* L.) increased significantly after the application of Fe_3_O_4_ pyrite NPs. A comparison between plants treated with Fe NPs and non-treated plants manifested better root growth in peanuts (*Arachis hypogaea* L.) (Rui et al., 2016) after the application of Fe NPs under field conditions. Moreover, according to Raju et al. (2016), green gram (*Vigna radiate* L.) depicts higher radical length and higher fresh biomass with Fe NPs application (2–6 nm) when compared to the non-treated condition (ferrous sulfate, Fe_3_SO_4_) during the germination stage. Movahhedy-Dehnavy et al. (2009) reported an 11% reduction in total dry matter (TDM) due to water deficit stress in the flowering stage. According to Sheykhbaglou et al. (2010), the application of nano-iron oxide at the concentration of 750 ppm caused an increase in dry weight in soybean plants under drought stress. Vaghar et al. (2020) reported that combining Fe and Zn foliar applications increased the TDM by 20.9 and 20.3%, respectively. According to Benzon et al. (2015), nano-fertilizers are always beneficial for plants, as they either supply nutrients or play an active role in the transportation or absorption of available nutrients, thus improving plant growth rate. Liu and Lal (2014) have also claimed a similar result that using phosphorus NPs increases the growth rate of soybeans by 33%. Nano-fertilizers can precisely release their active ingredients in response to environmental triggers and biological demands. In most cases, the plant height mainly depends on plant genetic characteristics and environmental conditions. So, under drought conditions, cell division and increase in cell size are difficult to achieve, which further prevents stem elongation and reduces plant height. Gholinezhad (2017) observed that nano-Fe_3_O_4_ fertilizer increased plant height and biomass dry yield by about 20% compared to the control (no fertilizer) condition. In particular, the use of Fe nano-fertilizer can increase plant growth, as the small and highly soluble compounds are generally absorbed quickly by plants which mitigate the food shortage and needs of plants (Rasht, 2013). Moreover, the availability of macro- and micronutrients in the Fe_3_O_4_ nano-fertilizers can be responsible for their growth-promoting activity.

Due to the application of nano-iron, chlorophyll levels of soybean plants increased significantly in our experiment. Our result was supported by Mohammadi et al. (2018), who suggested that the application of 750 ppm Fe_3_O_4_ nanoparticles as foliar spray increased the total chlorophyll content (0.55 mg g^−1^) of the peppermint plants. In another experiment, applying nano-Fe_3_O_4_ fertilizer positively affects peanuts and causes increased growth and photosynthesis. When compared to other treatments, such as organic materials and Fe_3_O_4_ citrate, nano-Fe_3_O_4_ fertilizer facilitated the transfer of photosynthate and Fe_3_O_4_ to the peanut leaves (Liu et al., 2005). In sunflower leaves, the total chlorophyll content increased positively after the application of biocompatible magnetic nanofluid (MNF) (Pirvulescua et al., 2014). After the exogenous application of nano-TiO_2_, the content of chlorophyll, carotenoids, and anthocyanin in barley also increased significantly (Janmohammadi et al., 2016). More specifically, chlorophyll structure, reception of sunlight, production of pigment, and RUBISCO activity all improved noticeably after the application of nano-TiO_2_, which further enhanced the amount of photosynthesis in the plant. In another study, chlorophyll content of spinach leaf increased by ~17 times, and simultaneously improved photosynthetic rate by 29% was also observed after the application of NTiO_2_ compared to those observed in the control plants (Gao et al., 2006). Also, according to Tarafdar et al. (2014), the chlorophyll content of pearl millet crops and savory plants can be increased with the foliar application of Zn nano-fertilizer.

The foliar application of nano-Fe_3_O_4_ fertilizer ensured higher water availability, and as a result, the water saturation deficit (WSD) was decreased substantially in our study. The minimum water saturation deficit was observed in 200 ppm nano-Fe_3_O_4_ fertilizer-treated plants, with only 22.73% in drought conditions and 22.71% in well-watered conditions. Taran et al. (2017) reported that Cu and Zn NPs induced an increase in RWC by 8–10% in the leaves of seedlings of two different wheat varieties exposed to drought stress. According to Deepa et al. (2015), compared to the regular relative water content (RWC) of plants (64%), the application of Fe and Cu NPs can maintain a higher relative water content (RWC) level of 71% in plants. For the transportation of nutrients and water content into the required region of the plant body, nano-fertilizers can perform reliably (Deepa et al., 2015). Eichert et al. (2008) discovered that engineered nanoparticles with a size of <50 nm could enter the stomatal pores of *Vicia faba* L. without any difficulty. Furthermore, Wang et al. (2013) found that 27.3–46.7 nm is the limit for watermelon for stomatal size exclusion. The exogenously applied nanoparticles are transported from the application site through phloem vessels and plasmodesmata (40 nm in diameter) to the heterotrophic cells (Knoblauch and Oparka, 2012). The bond between nanoparticles and carrier proteins permits the entry of nanoparticles into the plant cells using the pathways of aquaporin, ion channels, and endocytosis (Nair et al., 2010).

The positive effect of nano-iron on the number of seeds per plant in our results coincides with the reports of other researchers. The foliar application of Fe and Zn nano-chelates elevated the number of grains per plant by 31.2%. Under stress conditions, the flowering, podding, and seed filling of Fe and Zn nano-chelates increased the number of grains per plant more than other treatments. The application of Fe_3_O_4_ nanoparticles significantly improved the trait of 1,000-grain weight (19.86%) under drought stress (Sheykhbaglou et al., 2010). Sabet and Mortazaeinezhad (2018) reported that foliar application of nano-Fe_3_O_4_ significantly increased the grain yield due to direct absorption by leaves. The Fe_3_O_4_ nanoparticle significantly improved grain yield (37.43%), biological yield (22.91%), and harvest index (3.86%) under drought stress (Sheykhbaglou et al., 2010). Foliar application of Fe and Zn nano-chelates increased the average grain yield of soybean by 43.8% (Benzon et al., 2015). The sink strength of a plant can be defined as the capability of photosynthates to move from the source toward itself. The total biomass of the sink tissue, which is sinking size and photosynthates the uptaking rate of sink tissue per unit biomass known as the “sink activity” both the influencing factors of sink strength of plant (Taiz and Zeiger, 2006). According to our previous discussion, nano-fertilizers are responsible for the transportation and movement of various nutrients from roots to foliage and also for the movement of photosynthates all over the plants, which may affect the sink strength of plants. Due to this mechanism, yield and yield contributing traits can be increased after the application of nano-Fe_3_O_4_ fertilizers (Liu and Lal, 2014).

According to Chaves et al. (2009), increasing seed protein content under stress conditions was related to reducing the starch ratio to protein content due to the decrement of net photosynthetic rate. Mittler (2006) also agreed with this finding. He opined that under drought stress, the assimilation of CO_2_ was decreased; however, the remobilization of nitrogen from leaves to seeds did not decrease, so the protein content of seeds increased. However, in our experiment, the nano-Fe_3_O_4_ solution increased the protein content by 2.7 and 7.34% in drought and well-watered conditions, respectively, compared to the untreated plants (Table 6). The application of nano-fertilizers enhanced the protein content of bean seeds, and the highest content was observed in Fe + Zn treatment (FatollahpourGrangah et al., 2020). These results agree with Abd El-Aziz and Balbaa (2007), who reported that exogenously applied micronutrients, such as Fe and Zn, increased nitrogen percentage and protein content. Increasing seed protein content under a foliar spray of nano-fertilizer was related to increased content of soluble sugars (Bybordi and Mamedov, 1970), which serve as a source for synthesizing nitrogenous substances like amino acids. Also, Fe_3_O_4_ and zinc are two important constituents of enzymes involved in the biosynthesis of amino acids, thus protein content is increased under the foliar application of Fe and Zn nano-fertilizers (Sheykhbaglou et al., 2018).

Our results coincide with the result of Sakar et al. (1988) where the highest percentage of lipid was obtained by applying 750 ppm of nano-Fe_2_O_3_. According to the literature (Ravi et al., 2008), applying nano-iron also increases the lipid content of safflower. Vaghar et al. (2021) found the lowest oil content (17.47%) under drought conditions at the seed filling stage. In contrast, the highest oil content (23.73%) was obtained by the combined foliar application of nano-zinc and manganese in soybean. Davar et al. (2014) also reported that drought stress negatively impacts the yield components of safflower; however, oil percentage was mitigated through a foliar spray of iron nanoparticles (Fe NPs).

## Conclusion

Drought stress severely affected all physiological, morphological, and agronomic parameters in soybean plants when compared to those grown under well-watered conditions. On the other hand, foliar application of Fe_3_O_4_ nanoparticles reduced the negative effects of water deficiency. Under water deficit conditions, foliar application of 200 ppm Fe_3_O_4_ nanoparticles was more successful than other treatments in lowering oxidative stress by increasing the chlorophyll content, hydration status, seed oil, and protein content. These factors influenced plant dry matter accumulation, yield components, seed yield, and seed quality in a beneficial way. As a result of these findings, it was suggested that an exogenous foliar spray of Fe_3_O_4_ nanoparticles could be recommended to help soybean plants cope with drought stress conditions.

## Data availability statement

The original contributions presented in the study are included in the article/supplementary material, further inquiries can be directed to the corresponding authors.

## Author contributions

Conceptualization: MMan and DD. Original draft preparation: DD and US. Data curation: MMan. Visualization: MMan and US. Validation: US, MMam, MS, BA, OP, and RM. Reviewing and editing: US, TI, SE, MS, BA, OP, and RM. All authors have read and agreed to the published version of the manuscript.

## Funding

This work was supported by funds from the National Research Development Projects to Finance Excellence (PFE)-14/2022-2024 granted by the Romanian Ministry of Research and Innovation. This research was partially funded by UEFISCDI (Grant Number PN-III-P4-IDPCE-2020-2126).

## Conflict of interest

The authors declare that the research was conducted in the absence of any commercial or financial relationships that could be construed as a potential conflict of interest.

## Publisher's note

All claims expressed in this article are solely those of the authors and do not necessarily represent those of their affiliated organizations, or those of the publisher, the editors and the reviewers. Any product that may be evaluated in this article, or claim that may be made by its manufacturer, is not guaranteed or endorsed by the publisher.
